# Diagnostic system strengthening for drug resistant tuberculosis in Nigeria: impact and challenges

**DOI:** 10.4102/ajlm.v6i2.502

**Published:** 2017-03-31

**Authors:** Gambo Aliyu, Nicholas Ezati, Mosunmola Iwakun, Sam Peters, Alash’le Abimiku

**Affiliations:** 1 Institute of Human Virology, University of Maryland School of Medicine, Baltimore, Maryland, United States; 2 Institute of Human Virology, Abuja, FCT, Nigeria

## Abstract

**Background:**

The increasing prevalence of drug-resistant tuberculosis and the threat of extensively-drug-resistant tuberculosis in HIV hotspots have made the detection and treatment of drug-resistant tuberculosis in the sub-Saharan Africa setting a global public health priority.

**Objective:**

We sought to examine the impact and challenges of tuberculosis diagnostic capacity development for the detection of drug-resistant tuberculosis and bio-surveillance using a modular biosafety level 3 (BSL-3) laboratory in Nigeria.

**Method:**

In 2010, the United States President’s Emergency Plan for AIDS Relief (PEPFAR) programme, through the Institute of Human Virology at the University of Maryland in Baltimore, Maryland, United States, deployed a modular, BSL-3 laboratory to support the national tuberculosis programme in drug-resistant tuberculosis detection and bio-surveillance for effective tuberculosis prevention and control.

**Results:**

From 2010 until present, sputum samples from 11 606 suspected cases in 33 states were screened for drug-resistant tuberculosis. Of those, 1500 (12.9%) had mono-resistant tuberculosis strains, and 459 (4.0%) cases had multidrug-resistant tuberculosis. Over the last four years, 133 scientists were trained in a train-the-trainer programme on advanced tuberculosis culture, drug susceptibility testing, line-probe assays and Xpert^®^ MTB/RIF, in addition to safety operations for biosafety facilities. Power instability, running cost and seasonal dust are notable challenges to optimal performance and scale up.

**Conclusion:**

Movable BSL-3 containment laboratories can be deployed to improve diagnostic capacity for drug-resistant tuberculosis and bio-surveillance in settings with limited resources.

## Background

Nigeria has a limited high-level certified laboratory infrastructure and trained human resources to support a comprehensive public health response to the tuberculosis pandemic.^1^ Until recently, the conventional culture-based drug susceptibility testing (DST) platforms for detecting drug-resistant tuberculosis were available only in select public and private laboratories.^2^ Presumptive cases of multi-drug resistant (MDR) tuberculosis from different parts of the country went to the distant coastal city of Lagos for diagnosis and at the Nigerian Institute for Medical Research, which housed the only tuberculosis reference laboratory for a population of over 170 million. The number of culture reference laboratories has risen to seven, yet they covered only 4% to 8% of the World Health Organization-recommended population target of one functioning culture laboratory per 500 000 to one million population.^3^ According to a 2011 WHO report on global tuberculosis control, two in 100 of the newly detected tuberculosis cases and nine in 100 of re-treated cases in Nigeria were MDR tuberculosis.^4^ The National Tuberculosis and Leprosy Training Centre in Zaria has the largest tuberculosis referral center in northern Nigeria, with an average of 25–30 new smear-positive tuberculosis cases enrolled in treatment and care monthly; about 27% of the enrolled tuberculosis cases are co-infected with HIV.^5^ In addition to the reference laboratory, the centre has a large outpatient clinic for the management of tuberculosis, HIV and leprosy. It also has training facilities as the national training centre for tuberculosis and leprosy. It has a modest inpatient facility, mainly for the treatment of MDR tuberculosis, with a 20-bed capacity and two isolation rooms.

With over 80% of estimated tuberculosis cases currently undetected,^6^ the steady rise in case notification from 40 000 cases in 1999 to about 140 000 in 2010,^7^ coupled with increasing prevalence of drug-resistant tuberculosis and the potential threat of co-infection with HIV in patients with drug-resistant tuberculosis, underscore the need to strengthen diagnostic capacity for detection of tuberculosis and MDR tuberculosis in Nigeria.^8,9,10^ The scale-up of Cepheid GeneXpert^®^ for *Mycobacterium tuberculosis* and rifampicin resistance detection within a two-hour time frame requires complementary capacity for confirmatory DST with culture or line-probe assays. To enhance detection of tuberculosis, MDR tuberculosis and extensively-drug-resistant tuberculosis, as well as treatment monitoring across Nigeria, the establishment of biosafety level 3 (BSL-3) laboratories with capacity for culture and DST is a priority.

However, the few existing containment laboratories in Nigeria function suboptimally, with frequent breakdowns linked to design, structural complexities, adoptability and a harsh operational environment. To guarantee the optimal performance required of biosafety facilities to meet the unmet needs of the national tuberculosis programme, the Institute of Human Virology at the University of Maryland in Baltimore, Maryland, United States, developed a modular pre-constructed BSL-3 laboratory for the National Tuberculosis and Leprosy Training Centre in Zaria, Kaduna State, Nigeria, with a five-year contractual agreement for service and local staff training in BSL-3 laboratory operation and maintenance. In this article, we describe the establishment of the prototype of the modular BSL-3 laboratory as a platform for MDR tuberculosis surveillance in Nigeria and its impact on system strengthening and the challenges encountered.

## Conception, construction and assembly of the modular biosafety level 3 laboratory

**FIGURE 1:**
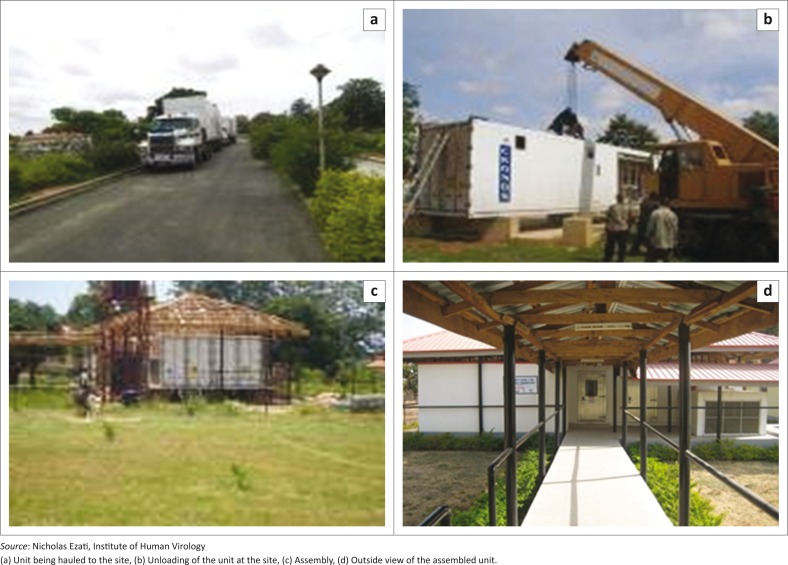
Delivery and assembly of the modular BSL-3 laboratory, Zaria, Kaduna State, Nigeria, 2010.

Following several failed attempts to upgrade or renovate existing structures and uncertainties about the structural building requirements to withstand the negative pressure required for such laboratories, Germfree Laboratories, Inc., based in Ormond Beach, Florida, United States, was contracted by the Institute of Human Virology at the University of Maryland to design, construct, deliver and assemble the movable BSL-3 laboratory with input from the Institute’s staff. Following several modeling sessions, the laboratory was designed using two 40-foot containers with all the equipment required to make it functional. It was then disassembled and the components and accessories were shipped to Lagos, Nigeria, and delivered to Zaria by oversized haulage truck to the National Tuberculosis and Leprosy Training Centre, where the laboratory is currently located (Figure 1).

**FIGURE 2:**
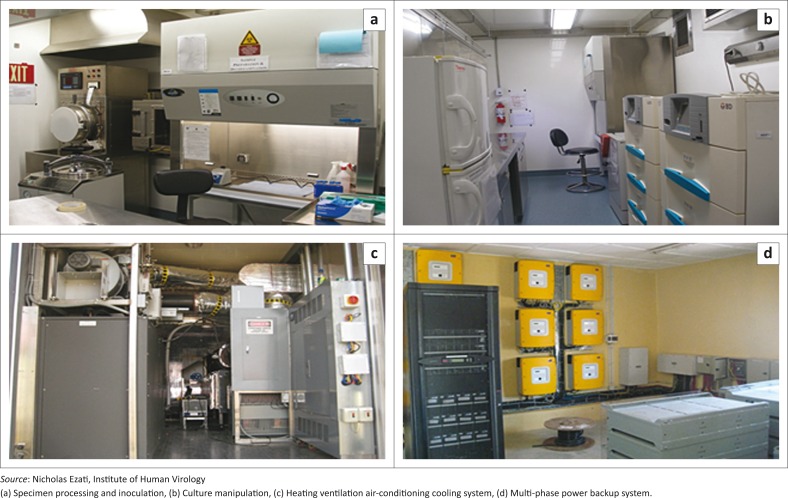
Sections of the modular BSL-3 laboratory, Zaria, Kaduna State, Nigeria, 2010.

The laboratory has the following sections: (a) an alcove; (b) a specimen receipt area with an autoclave and a pass-through-interlocked window where samples are received from outside, checked and registered; (c) a processing and inoculation section containing three biological hoods and a centrifuge; (d) a culture manipulation room with two BACTEC MGIT 960 (Becton Dickinson, Franklin Lakes, New Jersey, United States) instruments; and (e) a mechanical room. In the culture manipulation room, growth tubes with inoculated samples are grown in one BACTEC MGIT 960 instrument and DST is done in the other. The mechanical room houses all regulatory mechanisms, a heating, ventilation, air-conditioning, cooling control system and control panels for the operation of the laboratory, which can be viewed remotely from Germfree Laboratories in Florida (Figure 2). This is used for monitoring the functionality of the laboratory, and for the training and mentoring of Nigerian biotech engineers. Laboratory scientists from different parts of country receive trainings on good laboratory practices, assays, advanced culture with DST, and handling of hazardous materials.

Two backup generators, a 250 and a 100 KVA, are used to supplement the public power supply to provide a 24-hour seven-day supply of electricity. The heating, ventilation, air-conditioning, cooling system and all equipment are further supported through a 60 KW and 30 KVA three-phase online power inverter system. To keep rodents out, a mesh fence was built around the laboratory and fumigation was performed.

The estimated cost for the modular BSL-3 laboratory, including equipment, delivery and the concrete platform upon which the lab rests, was $720 000. The two power generators cost $70 105, while the walkways, overhead water tanks, emergency showers, closed-circuit television system and other structural work cost $80 749. The sum of the estimated expense for the establishment of the modular BSL-3 laboratory was $870 854.

## Impact: system strengthening and drug-resistant tuberculosis diagnostic capacity

**FIGURE 3:**
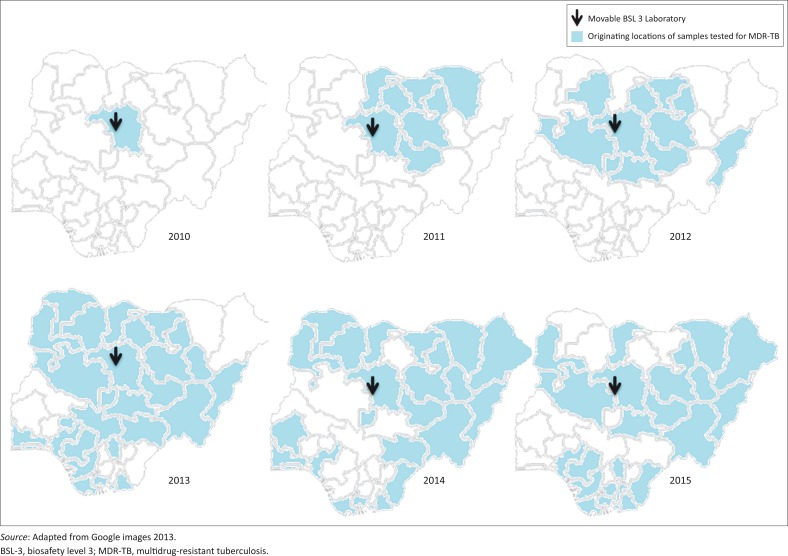
Sample referral trends in different parts of Nigeria for drug-resistant tuberculosis testing at the modular BSL-3 laboratory, Zaria, Kaduna State, Nigeria, 2010.

The modular laboratory serves as one of the two tuberculosis national reference laboratories in Nigeria. Services were mostly limited to the northern states until 2013, but by 2015 both northern and southern states were served (Figure 3). The laboratory performs and builds in-country capacity to perform solid and liquid cultures, DST, line-probe assays and GeneXpert tests. The line-probe assay and GeneXpert tests are not performed in the BSL-3 laboratory, but in a separate laboratory in the same complex by the same laboratory personnel.

A total of 89 scientists have received training on advanced tuberculosis culture, drug susceptibility testing and line-probe assays, and 44 scientists from nine states have been trained on GeneXpert. These include state external quality assurance officers and programme staff responsible for activating new GeneXpert sites, monitoring and supervision. The trained scientists from zonal tuberculosis reference laboratories now perform these tests in their respective laboratories and train their colleagues. The BSL-3 laboratory is also used for the preparation of external quality assurance panels for GeneXpert tests, line-probe assays and tuberculosis culture and DST tests across the country. Over the period from January 2010 to April 2016, sputum samples from 11 606 presumptive drug-resistant tuberculosis cases in 33 states were processed using solid and liquid cultures, then DST was performed using line-probe assays or solid proportion and BACTEC MGIT 960 methods. Of those, 1500 (12.9%) had mono-resistant strains, while 459 (4.0%) cases had MDR tuberculosis. The laboratory currently collaborates with the San Rafael World Health Organization supranational reference laboratory in Milan, Italy, the National Institute of Communicable Diseases in South Africa, and the Uganda Supranational Tuberculosis Reference Laboratory on external quality assessment, and is enrolled in the World Health Organization Regional Office for Africa’s Strengthening Laboratory Management Towards Accreditation step-wise accreditation programme with support from the African Society for Laboratory Medicine and the Medical Laboratory Science Council of Nigeria. The laboratory supported the 2010–2011 national prevalence survey for MDR tuberculosis and the 2012 tuberculosis prevalence survey in Nigeria.

## Barriers encountered in the process of establishing and maintaining biosafety level 3 laboratory services in Nigeria

As in most developing countries, an erratic power supply is a major challenge in Nigeria. The modular BSL-3 laboratory was operated on stand-by generators supported with a high capacity inverter system to ensure a steady supply of electricity. This added to the cost of operationalising the laboratory. The dry Harmattan wind from the Sahara Desert generates a lot of dust in winter in the north of Nigeria, where the laboratory is located. Germfree Industries, Inc. designed a pre-filtration unit using easily replaceable filters to filter the air supply before it reaches the more costly high-efficiency particulate air supply filters of the heating, ventilation and air conditioning system.

The public water supply at the site was infrequent and insufficient for laboratory use at the time of installation. An overhead tank was designed specifically to supplement the public water supply for the laboratory. A water filter was attached to ensure that clean water reached the distiller. Waste disposal was a concern and was addressed by passing liquid waste through a pre-treatment system before it is deposited into a septic tank. Spillage incidents at the operational training sessions necessitated the provision of an additional emergency shower to enhance personal protection. Access to the shower had to be fabricated to link it to the two emergency doors of the laboratory.

## Discussion

There are few BSL-3 tuberculosis laboratory prototypes in the sub-Saharan African setting. The modular BSL-3 laboratory offers a useful alternative for construction of such a facility. Lack of indigenous firms with professional expertise in the construction and maintenance of a BSL-3 containment laboratory to international standards – which is a critical first step in establishing a biosafety laboratory^11^ – informed the decision to buy a pre-constructed model. A modular laboratory was deployed by ZAMSTAR in 2009 in Zambia to conduct a national prevalence survey. Like our model, it was constructed in a 40-foot container with all equipment and accessories.^12^ However, compared with the ZAMSTAR model, our model cost more, because of additional accessories including power backups, cameras, walk ways, and overhead water tanks required for optimal functioning of the laboratory. Although the establishment of this laboratory appears to be expensive in the beginning, given the unmet need for expertise in biosafety containment and development of local capacity, it is an investment worth the cost. During the five years after its instalment, operations have been optimised to meet the local needs of the national tuberculosis programme without a break in service delivery.

With support from our modular BSL-3 laboratory, Nigeria was able to conduct national tuberculosis surveillance on a representative sample with best estimates of tuberculosis and drug-resistant tuberculosis prevalence. The scientists trained in tuberculosis detection and DST from various zonal laboratories that form the country’s tuberculosis laboratory network are now supporting tuberculosis diagnosis and treatment monitoring of patients in their respective zones. This capacity development programme has helped expand the number of laboratories supporting the tuberculosis control programme from three in 2010 to seven in 2016. With the increasing demand for services resulting from the scale-up of innovative strategies for intensified case finding, this investment is expected to contribute to epidemic control by improving early detection and monitoring of treatment of drug-resistant tuberculosis in Nigeria.

Elsewhere in Africa, locally constructed BSL-3 laboratories are able to operate optimally.^13,14^ However, in many settings within sub-Saharan Africa, the experience and expertise of local construction workers may not guarantee internationally-accepted standards for biosafety containment facilities.^11,13^ To further guarantee standards and prevent failures experienced on previous attempt to integrate such services in locally constructed laboratories, the PEPFAR programme entered into a service contract with Germfree Laboratories, Inc. This allowed the local team of biomedical engineers, who would be shouldered with the responsibility of maintaining the unit for the long term, to work closely with the Germfree team for a brief period after the installation. Now that the unit is locally maintained, Germfree only performs annual certification and preventive maintenance. The laboratory is linked to suprareference laboratories for external quality assessment.

Our experience provides evidence that it is feasible to establish and maintain modular containment laboratories in settings with limited resources. International donor agencies and policy makers should consider replicating this model in high tuberculosis-burden settings, especially in places where expertise in construction and supervision of BSL-3 facilities are not locally available, as demonstrated in the case of Nigeria. These laboratories are easy to integrate into national tuberculosis programs and serve as training facilities for capacity development.

Upgrading an existing tuberculosis laboratory to a BSL-3 standard for detection of drug-resistant tuberculosis, as was successfully done in the Kingdom of Lesotho, is another alternative way of providing the needed diagnostic services.^14^ If that is considered, the experience and expertise of the construction firm is necessary to guarantee internationally-acceptable standards.

## Conclusion

A modular BSL-3 laboratory was deployed to strengthen detection of tuberculosis and drug-resistant tuberculosis in Nigeria. It operates optimally and collaborates effectively with established international laboratories to strengthen the national tuberculosis programme. Priority should be given to modular laboratories in settings with limited expertise in building facilities for the isolation and characterisation of dangerous biological agents such as drug-resistant and MDR tuberculosis. Since the establishment of this first prototype model in a developing country setting, Germfree Laboratories, Inc. has used the knowledge acquired to establish a modified and more affordable biosafety level 2 system that has the capacity for BSL-3 practices with the addition of a Class III biosafety cabinet, also referred to as glove box. The addition of the Class III biosafety cabinet with a direct sample entry pass-box makes it possible to safely handle highly-infectious samples.

BOX 1 Lessons Learned.

- It is feasible to establish and maintain tuberculosis diagnostic and containment laboratories in settings with limited expertise
- Such model laboratories can support in-country capacity building for diagnostic system strengthening
- Such laboratories are suitable where local expertise in the construction and maintenance of biosafety level 3 containment laboratories according to international standards is lacking
